# The physiological role of TRP channels in sleep and circadian rhythm

**DOI:** 10.1111/jcmm.18274

**Published:** 2024-04-27

**Authors:** Geoffrey Woodard, Juan A. Rosado, He Li

**Affiliations:** ^1^ Department of Psychiatry Uniformed Services University of Health Sciences Bethesda Maryland USA; ^2^ Department of Physiology University of Extremadura Caceres Spain

**Keywords:** circadian rhythm genes, clock (*Clk*), period 1 (*Per1*), period 2 (*Per2*), suprachiasmatic nucleus (*SCN*), timeless (*tim*), TRP channels, sleep, TRPA1, TRPV1

## Abstract

TRP channels, are non‐specific cationic channels that are involved in multiple physiological processes that include salivation, cellular secretions, memory extinction and consolidation, temperature, pain, store‐operated calcium entry, thermosensation and functionality of the nervous system. Here we choose to look at the evidence that decisively shows how TRP channels modulate human neuron plasticity as it relates to the molecular neurobiology of sleep/circadian rhythm. There are numerous model organisms of sleep and circadian rhythm that are the results of the absence or genetic manipulation of the non‐specific cationic TRP channels. *Drosophila* and mice that have had their TRP channels genetically ablated or manipulated show strong evidence of changes in sleep duration, sleep activity, circadian rhythm and response to temperature, noxious odours and pattern of activity during both sleep and wakefulness along with cardiovascular and respiratory function during sleep. Indeed the role of TRP channels in regulating sleep and circadian rhythm is very interesting considering the parallel roles of TRP channels in thermoregulation and thermal response with concomitant responses in growth and degradation of neurites, peripheral nerves and neuronal brain networks. TRP channels provide evidence of an ability to create, regulate and modify our sleep and circadian rhythm in a wide array of physiological and pathophysiological conditions. In the current review, we summarize previous results and novel recent advances in the understanding of calcium ion entry via TRP channels in different sleep and circadian rhythm conditions. We discuss the role of TRP channels in sleep and circadian disorders.

## 1 INTRODUCTION

Regulation of the activation of transient receptor potential (TRP) channels could lead to the development of new treatments for numerous sleep and circadian rhythm disorders. A lot of scientific evidence has shown the molecular role of TRP channels in regulating neuronal networks, peripheral nerves and communication between various regions of the brain that regulate sleep and circadian rhythm. Combined, TRP channels play a regulatory role that is controlled by the environmental factors that effect TRP channels. Regulatory control of sleep and circadian rhythm through TRP channels shows how the brain is dynamically modified on a cellular level based on environmental experiences that can then again be reshaped for better or for worse. Here we look at various sleep and circadian disorders and how they are regulated on a cellular and molecular level by TRP channels. These findings, in turn, reinforce neuroscientists to perceive efficacious therapeutics that support these biological explanations of how sleep and circadian rhythm is regulated.

## 2 OVERVIEW OF THE MAMMALIAN TRP CHANNEL FAMILY

TRP proteins are six transmembrane domain‐containing subunits that form homo‐ or heterotetrameric non‐selective cation channels. The TRP superfamily includes at least 28 related channels that play an important role in several cellular functions ranging from sensory transduction (including invertebrate vision, temperature, pain and gustatory and osmolarity detection) to development. The first member of the TRP superfamily was identified as a protein involved in phototransduction in Drosophila.^1^ The *trp* gene was named on the basis of the transient, rather than sustained, receptor potential observed in response to light in mutant flies. From the beginning a relationship between TRP proteins and ionic currents across the membrane was suggested since TRP mutants displayed a defect in light‐induced Ca^2+^ influx, which together with the predicted structure of TRP and the related protein, TRPL, raised the possibility that these proteins were Ca^2+^ influx channels.^2^, ^3^

TRP proteins are present in yeast, *Drosophila*, *Caenorhabditis elegans*, fish and mammals. TRP channels are widely expressed in both excitable and non‐excitable cells, where they have been reported to mediate Ca^2+^ entry. Although all TRP proteins form cation channels, they differ significantly in their cation permeability and activation mechanisms, although most members of the TRP superfamily share significant sequence homology.

TRP channels can be grouped into six subfamilies: those most closely related to *Drosophila* TRP (TRPC, TRPV and TRPM), two subfamilies that are more distantly related to *Drosophila* TRP (TRPP and TRPML), and a less related TRPN group that is absent in mammals but expressed in flies and worms and includes the mechanosensory channel NOMPC.^2^, ^3^ The TRPC subfamily groups the mammalian proteins that display the greatest similarity to *Drosophila* TRP, sharing between 32% and 47% amino acid homology over the N‐terminal region. TRPC proteins show the prototypical structure, including three or four ankyrin repeats, the six transmembrane domains, and a highly conserved 25 amino acid sequence known as the TRP box, a hydrophobic region located just C‐terminal to the sixth transmembrane domain. TRPV proteins also include three or four ankyrin domains as well as the TRP box, and TRPM proteins contain a TRP box, but no ankyrin repeats, and some members, such as TRPM6 and TRPM7 exhibit a C‐terminal kinase domain.^3^

Most TRP channels are non‐selective for monovalent and divalent cations with wide range of Ca^2+^ to Na^+^ permeability ratios. Especially relevant are TRPM4 and TRPM5, which are selective for monovalent cations, as well as the Ca^2+^‐selective members TRPV5 and TRPV6 that exhibit a Ca^2+^ to Na^+^ permeability ratio over 100. Ca^2+^ and Na^+^ influx through TRP channels leads to membrane depolarization while increasing cytosolic Ca^2+^ and/or Na^+^ concentrations,^4^ thus reducing the driving force to Ca^2+^ influx to other Ca^2+^ channels. This article presents an overview of what is currently know of the molecular relationship between TRP channels and their role in physiological cell processes that regulate sleep and circadian rhythm.

## 3 TRP CHANNELS REGULATE SLEEP AND CIRCADIAN RHYTHM BY SENSING LIGHT AND HEAT

Temperature and light modulate our circadian rhythm by a mechanism involving TRP channels and clock genes.^5^, ^6^ The light information recorded through the retina is translated into the physiological circadian response,^7^, ^8^, ^9^, ^10^ thus, the organism is able to respond to differences in environment light and temperature to maximize cellular energy and metabolic resources efficiently throughout the day.^11^, ^12^ It was shown that temperature plays a highly significant role in regulating our sleep and circadian rhythm through multiple clock genes and TRP channels.^13^ The modulation of the circadian rhythm by temperature is mediated by a mechanism involving the activation of rhodopsin and TRP channels.^14^ This is a molecular mechanism whereby external light and temperature are able to communicate sleep and wakefulness through multiple clock genes^15^ and TRP channels located throughout the body including muscle cells, neuronal cells and peripheral nerves sending cues as to time of day.^16^, ^17^ The molecular signalling mechanism of circadian rhythm and sleep in mammals works through light (photons) activating photoreceptors (rhodopsin) that in turn connect with the central nervous system activating multiple synapses.^18^, ^19^, ^20^, ^21^, ^22^, ^23^, ^24^, ^25^, ^26^ Meanwhile, temperature information allows TRP channels to make modifications in the response of clock genes in the human body.^18^, ^24^, ^26^, ^27^, ^28^, ^29^, ^30^, ^31^ Indeed, light sensing retina expresses TRPC6^10^, ^32^, ^33^, ^34^ and TRPC7,^33^, ^34^ whereas, changes in heat are sensed by TRPV1^35^, ^36^, ^37^, ^38^, ^39^, ^40^, ^41^, ^42^ and changes in cold are sensed by TRPM8.^12^, ^36^, ^43^ The physiological response to heat and light is often referred to as entrainment that derives from the French word entrainer, meaning to ‘bring on as a consequence’ or to ‘drag in’.^44^, ^45^, ^46^, ^47^, ^48^, ^49^ Our physiological response of sleep and wakefulness are a direct response to our environmental conditions of light and temperature.^50^, ^51^, ^52^ Changes in heat, that are similar to changes found in human body temperature were able to switch the *Per2* and *Clock* genes on and off in NIH3T3 fibroblasts in culture.^53^, ^54^, ^55^, ^56^ The suprachiasmatic nucleus, located in the hypothalamus, is responsible for regulating the effects of temperature change upon sleep and the circadian rhythm.^57^ Despite suprachiasmatic nucleus‐projecting retinal ganglion cells act independently and separately from photoreceptors, the retinal ganglion cells express TRP channels which behave similarly as in photoreceptors upon light stimulation.^58^ Indeed, it has been hypothesized that the modulation of the circadian rhythm is not solely delegated to photoreceptors. The period (*Per*) and timeless (*tim*) genes have been shown to regulate circadian rhythm and sleep in *Drosophila*.^59^ *NinaE* fruit flies that genetically lack the gene and protein (rh1) for the rhodopsin receptors was able to show sleep, wakefulness and circadian rhythm behaviour in the absence of photoreceptors.^59^ In addition, when *Drosophila* flies were mutated for *trp* and *trpl* genes their visual transduction cascade was impaired, which attenuated the *tim* gene response to light, but circadian rhythm behaviour was only partially disturbed, thus suggesting that the circadian rhythm does not rely on the visual system but has its own independently dedicated system for photoreception.^59^

## 4 TRP CHANNELS REGULATING METABOLISM PLAY A ROLE IN SLEEP AND CIRCADIAN RHYTHM

Orexins, particularly Orexin A and Orexin B, have been shown to be involved not only in insulin secretion and energy metabolism, but also in the sleep–wake circadian rhythm cycle. It has been reported that blockade of TRP channel‐mediated calcium release by lanthanum abrogates orexin activity, thus suggesting that TRP channels are involved in the mediation of orexin functions.^60^ Indeed, fat metabolism has been shown to play a role in regulating the circadian rhythm. TRP channels have been shown to regulate the circadian rhythm, sleep wake cycle, along with fat and energy metabolism in brown adipose tissue.^12^ TRPM8, known as a cold temperature sensing receptor, was found to regulate the clock circadian rhythm genes in brown adipose tissue along with the circadian rhythm gene *Per1*. Clock and Per1 genes amplitude and oscillation were found to be reduced both in the eyes and brown adipose tissue of TRPM8 knockout mice. Similarly, UCP1, a mitochondrial membrane protein essential in brown fat metabolism was greatly reduced.^12^ Heat sensitive TRPV1 receptors were shown to alter matrix metalloprotein expression independent of clock genes in the eye.^36^ TRPA1 was found to regulate sleep and circadian rhythm in response to environmental temperature.^61^ TRPA1 was the first thermosensing TRP channel to be described in invertebrates associated to the regulation of the rhythmic sleep–wake changes in body temperature: cooler when sleeping and warmer when awake.^61^ Melanopsin receptors, that regulate the duration of the sleep–wake cycle in response to light in certain organisms, have been shown to mediate their effects on the sleep–wake cycle through TRP channels activation, particularly in amphioxus.^62^ Temperature sensing TRP channels have been shown to environmentally synchronize the cold‐warm body temperature changes that correspond with the sleep–wake circadian rhythm cycle. *Drosophila* flies lacking the *Pyrexia* gene, a TRP channel found in the fruit fly, have been found to be unable to synchronize their behaviour to temperature cycles in the lower range (between 16 and 20°C), which provide further evidence for the involvement of TRP channels in the synchronization of the circadian rhythm by temperature.^63^

## 5 TRP CHANNELS ASSOCIATION WITH SLEEP AND CIRCADIAN RHYTHM GENES

The TRP channel, TRPA1, was shown to control arousal from sleep as TRPA1 knockout mice lack of arousal from sleep when exposed to noxious formalin odours.^64^ The circadian rhythm in *Drosophila* regulates colour discrimination and preference with TRPA1 controlling the preference of dim light over the colour green during the midday and avoidance of blue light during the day, which is controlled through rhodopsin 7 and the *Drosophila* TRP channel Painless found in multi‐dendritic neurons.^65^

A role for TRP channels in bladder function has also been described to be regulated by clock genes. An increase in gene expression for *TRPV1*, *TRPV4*, *Piezo1*, and *VNUT* in the spontaneously hypertensive rat (SHR) was attributed to having a regulatory role over circadian genes *Cry2* and *Clock* in the SHR bladder resulting in greater number of urination times during day and night cycles but a lower urination volume.^66^ Bladder function, including frequency of urination, day or night occurrence of urination and volume, are all regulated by circadian clock genes such as *Per2*. One interesting study looked at the circadian gene regulation of bladder function under conditions of stress. A drug that inhibits Per2 phosphorylation, PF670462 (PF), was able to correct irregular stress‐induced clock gene expression along with sensory bladder fullness genes, such as TRPV4, Piezo1 and Connexin26 in restoring normal circadian gene control of bladder function.^67^ Another study has reported that the expression of mechanosensory, such as Piezo1 and TRPV4, and main ATP release pathways, including Connexin26 and vesicular nucleotide transporter (VNUT), are regulated by clock genes in the bladder mucosa, thus, the expression of these genes is low in the sleep phase and modulating the frequency of urination during sleep.^68^

It has been reported that surgical removal of the mutant temperature sensing *TRPA1* gene called Pyrexia or *Pyx*, from the antennae of *Drosophila* restores normal circadian rhythm of mechanosensory neurological function of body positioning (proprioception) and hearing through the circadian protein called PERIOD.^69^ *Drosophila* fruit fly has 13 different TRP channels, 9 that are directly involved in regulating the circadian rhythm cycle. Among them, TRP, TRPL, Inactive, Brivido‐1, Brivido‐2, Brivido‐3 are all *Drosophila* TRP channels that play a circadian rhythm role in thermotaxis and locomotion in direct reaction to cool temperatures, whereas, the dTRPA1 provides circadian rhythm locomotion response to warmer temperatures, fluctuation in temperature and avoiding toxic or noxious heat. Finally, Painless and Pyrexia TRP channels are involved in noxious heat avoidance and regulating the circadian rhythm cycle in response to fluctuations in temperature.^70^ *Drosophila* neurons that express the heat sensing TRP channel, dTRPA1 have been shown to regulate motor activity of the sleep/wake cycle corresponding to light, day/night cycle, in fruit flies.^71^ The diurnal/daily intestinal motor activity found in gastrointestinal reflux disease was shown to be regulated both by TRPV1 channels along with the circadian rhythm genes *Per1*, *Per2*, *BMAL1* and *CRY2*, *TRPV1*, along with *NGF*.^72^ Recently, it was discovered that during the afternoon, not just morning or evening (siesta behaviour), locomotive behaviour such as seeking a shaded location from the warm sun, is also regulated by the *Drosophila* heat thermosensing dTRPA1 channel.^73^ Neonatal rats that were treated with capsaicin had TRPV1 receptors desensitized to heat but had an inverse to normal circadian rhythm body temperature cycle and circadian, *Hsf1* and *Per2*, gene expression.^37^ Mutant TRPA1 *Drosophila* were shown to have shorter morning activity with evening activity occurring later than normal under circadian cycle light conditions and at 18 degrees centigrade.^74^ The thermosensing TRP receptor Pyrexia was found in peripheral sensing chordotonal organs of *Drosophila* where they synchronize temperature with the circadian rhythm clock genes period (*Per*) and timeless (*tim*) forming a negatively regulated feedback loop with circadian transcription factors clock (*clk*) and cycle (*cyc*).^63^ *Drosophila* TRP channel, TRPA1 is found in the pacemaker neurons of the *Drosophila* brain where it is found to regulate the 2–3°C decline in temperature during the circadian sleep/wake cycle of the fruit fly.^75^ Similar to TRP channels, another calcium permeable ion channel located in the suprachiasmatic nucleus of the brain are the hyperpolarization‐activated cyclic nucleotide‐gated (HCN) channels that have also been shown to regulate the circadian rhythm gene *Per2*.^76^ The cytokine IL‐15 in the hypothalamus has been reported to regulate both metabolism and temperature in a circadian rhythm fashion in participation with TRPV4.^38^ Likewise, somatostatin has been shown to alter the function of nucleotide gated ion channels in their response to circadian rhythm genes and light.^77^

## 6 THE ROLE OF TRP CHANNELS IN SLEEP APNEA

TRP channels have also been found to be involved in sleep apnea. TRPC5 has been found to be highly elevated in obstructive sleep apnea suggesting that calcium entry through this channel might play a role in the myocardial damage that occurs in obstructive sleep apnea.^78^ Inflammatory mediator regulation of TRP channels by circulating exosomal miRNAs has been shown to play an important role in abnormal circadian regulation of blood pressure and could also serve as an indicator of the risk of cardiovascular disease associated with obstructive sleep apnea.^79^ Sleep deprivation has been shown to cause dry eye by producing unusual microvilli formation in superficial corneal epithelial cells that has been linked to low levels of TRPV6 expression.^80^ External environmental temperature has been shown to play a large role in regulating sleep duration and sleep circadian rhythm behaviour through the *Drosophila* TRPA1 channels located in the neuronal circuits.^61^ N‐acyltaurine is a fatty acid amide that activates and acts as an agonist for TRP channels. Fatty acid amide hydrolase (FAHH) is an enzyme that hydrolyses fatty acid amides. N‐arachidonoyl‐serotonin (AA‐5‐HT) is a TRPV1 channel blocker that is shown to alter the sleep and circadian rhythm cycle when administered at the start of the dark period resulting in a lack of wakefulness and heightened slow wave sleep along with an increase in the rapid eye movement sleep phase.^81^ TRP channels have been shown to be upregulated in the tobacco hornworm, *Manduca sexta* during the quiescent state characteristic of the moult.^82^ Obstructive sleep apnea was shown to increase the sensitivity of posterior cerebral arteries to the vasoconstrictor endothelin‐1 through elevated endothelin‐B receptor activity, and increased activation of TRPC receptors and Rho kinase. Excessive vasoconstriction of posterior cerebral arteries associated to obstructive sleep apnea was alleviated through use of the TRPC receptor antagonist SKF96365.^83^ Likewise, elevated expression of ion channel proteins was found in chronic obstructive sleep apnea with remodelling of the cardiac atrium.^84^

## 7 ROLE OF TRP CHANNELS IN CONTROLLING ALERTNESS AND PREVENTING SLEEP

TRPA1 knockout mice were found to be unresponsive in the fight or flight response noxious formalin odours. In fact, these mice were shown to sleep completely through exposure to toxin odours that were found to have caused massive effect on the brain as measured by c‐fos expression^64^ Spinal D‐amino acid oxidase was shown to induce sleep derived mechanical pain sensitivity through production of hydrogen peroxide, a direct pain inducing agonist of the TRPA1 nociceptive receptor.^85^ Studies expressing the temperature‐gated TRPA1 in *Drosophila* neurons to induce sleep on demand have reported that sleep facilitate consolidate memory in *Drosophila*.^86^ Recently it was shown that peripheral sensory organs contribute to temperature synchronization of the circadian clock in a cell autonomous mechanism that involves TRP channels^87^ TRPM4 channels were found to be expressed in circadian associated pacemaker LC and SCN neurons, and that TRPM4 contributes to subthreshold oscillations observed in those cells in neonatal mouse brainstem slices.^88^

TRP channels, as described in this review, play a significant role in regulating activity revolved around the quality and duration of sleep corresponding to the circadian rhythm cycle. TRP channels likewise form a systemic neurosensory chain orchestrating the relationship between sleep and circadian rhythm. TRP channels clearly show that sleep is important for long‐term memory and that even memory and control of temperature regulation during sleep along with memory of pain that effects sleep and wakefulness all intricately depend on coordination with TRP channels.

## AUTHOR CONTRIBUTIONS

**Geoffrey Woodard:** Writing – original draft (lead); writing – review and editing (lead). **He Li:** Conceptualization (lead); supervision (lead); writing – original draft (supporting); writing – review and editing (supporting). **Juan A. Rosado:** Writing – review and editing (equal).

## CONFLICT OF INTEREST STATEMENT

The authors declare no conflicts of interest.

## Data Availability

Data sharing is not applicable to this article as no new data were created or analyzed in this study.

## References

[1] Montell C , Rubin GM . Molecular characterization of the Drosophila trp locus: a putative integral membrane protein required for phototransduction. Neuron. 1989;2(4):1313‐1323. 2516726 10.1016/0896-6273(89)90069-x

[2] Montell C , Birnbaumer L , Flockerzi V . The TRP channels, a remarkably functional family. Cell. 2002;108(5):595‐598. 11893331 10.1016/s0092-8674(02)00670-0

[3] Montell C , Birnbaumer L , Flockerzi V , et al. A unified nomenclature for the superfamily of TRP cation channels. Mol Cell. 2002;9(2):229‐231. 11864597 10.1016/s1097-2765(02)00448-3

[4] Clapham DE . TRP channels as cellular sensors. Nature. 2003;426(6966):517‐524. 14654832 10.1038/nature02196

[5] Liu J , Liu W , Thakur D , Mack J , Spina A , Montell C . Alleviation of thermal nociception depends on heat‐sensitive neurons and a TRP channel in the brain. Curr Biol. 2023;33(12):2397‐2406 e6. 37201520 10.1016/j.cub.2023.04.055 PMC10330845

[6] Wang Z , Li S , Yao JW , et al. Differential expressions of hypothalamic thermosensitive TRP ion channels may underlie the posthatching ontogeny of brain cooling capacity in broiler chickens. Poult Sci. 2023;102(8):102782. 37276706 10.1016/j.psj.2023.102782 PMC10258507

[7] Richter M , Fingerhut BP . Regulatory impact of the C‐terminal tail on charge transfer pathways in Drosophila cryptochrome. Molecules. 2020;25(20):1‐14. 10.3390/molecules25204810 PMC7587983 33086760

[8] Immonen EV , French AS , Torkkeli PH , Liu H , Vähäsöyrinki M , Frolov RV . EAG channels expressed in microvillar photoreceptors are unsuited to diurnal vision. J Physiol. 2017;595(16):5465‐5479. 28087896 10.1113/JP273612 PMC5556157

[9] Pulido C , Malagón G , Ferrer C , et al. The light‐sensitive conductance of melanopsin‐expressing Joseph and Hesse cells in amphioxus. J Gen Physiol. 2012;139(1):19‐30. 22200946 10.1085/jgp.201110717 PMC3250099

[10] Warren EJ , Allen CN , Brown RL , Robinson DW . The light‐activated signaling pathway in SCN‐projecting rat retinal ganglion cells. Eur J Neurosci. 2006;23(9):2477‐2487. 16706854 10.1111/j.1460-9568.2006.04777.x PMC2435203

[11] Alachkar A . Aromatic patterns: tryptophan aromaticity as a catalyst for the emergence of life and rise of consciousness. Phys Life Rev. 2022;42:93‐114. 35905538 10.1016/j.plrev.2022.07.002

[12] Moraes MN , de Assis LVM , Henriques FS , Batista Jr ML , Güler AD , Castrucci AML . Cold‐sensing TRPM8 channel participates in circadian control of the brown adipose tissue. Biochim Biophys Acta, Mol Cell Res. 2017;1864(12):2415‐2427. 28943398 10.1016/j.bbamcr.2017.09.011

[13] Buhr ED , Yoo SH , Takahashi JS . Temperature as a universal resetting cue for mammalian circadian oscillators. Science. 2010;330(6002):379‐385. 20947768 10.1126/science.1195262 PMC3625727

[14] Shen WL , Kwon Y , Adegbola AA , Luo J , Chess A , Montell C . Function of rhodopsin in temperature discrimination in drosophila. Science. 2011;331(6022):1333‐1336. 21393546 10.1126/science.1198904

[15] Guido ME , Monjes NM , Wagner PM , Salvador GA . Circadian regulation and clock‐controlled mechanisms of glycerophospholipid metabolism from neuronal cells and tissues to fibroblasts. Mol Neurobiol. 2022;59(1):326‐353. 34697790 10.1007/s12035-021-02595-4

[16] Franzago M , Alessandrelli E , Notarangelo S , Stuppia L , Vitacolonna E . Chrono‐nutrition: circadian rhythm and personalized nutrition. Int J Mol Sci. 2023;24(3):1‐17. 10.3390/ijms24032571 PMC9916946 36768893

[17] Rajput P , Kumar D , Krishnamurthy S . Chronic exposure to dim artificial light disrupts the daily rhythm in mitochondrial respiration in mouse suprachiasmatic nucleus. Chronobiol Int. 2023;40:1‐14. 37483020 10.1080/07420528.2023.2236708

[18] Nayak G , Zhang KX , Vemaraju S , et al. Adaptive thermogenesis in mice is enhanced by opsin 3‐dependent adipocyte light sensing. Cell Rep. 2020;30(3):672‐686. e8. 31968245 10.1016/j.celrep.2019.12.043 PMC7341981

[19] Kronauer RE , St Hilaire MA , Rahman SA , Czeisler CA , Klerman EB . An exploration of the temporal dynamics of circadian resetting responses to short‐ and long‐duration light exposures: cross‐species consistencies and differences. J Biol Rhythm. 2019;34(5):497‐514. 10.1177/0748730419862702 PMC7363039 31368391

[20] Lichtenstein L , Grubel K , Spaethe J . Opsin expression patterns coincide with photoreceptor development during pupal development in the honey bee, Apis mellifera. BMC Dev Biol. 2018;18(1):1. 29382313 10.1186/s12861-018-0162-8 PMC5791347

[21] Paschos GK , Ibrahim S , Song WL , et al. Obesity in mice with adipocyte‐specific deletion of clock component Arntl. Nat Med. 2012;18(12):1768‐1777. 23142819 10.1038/nm.2979 PMC3782286

[22] Munch M , Léon L , Crippa SV , Kawasaki A . Circadian and wake‐dependent effects on the pupil light reflex in response to narrow‐bandwidth light pulses. Invest Ophthalmol Vis Sci. 2012;53(8):4546‐4555. 22669721 10.1167/iovs.12-9494

[23] Meesters Y , Dekker V , Schlangen LJM , Bos EH , Ruiter MJ . Low‐intensity blue‐enriched white light (750 lux) and standard bright light (10,000 lux) are equally effective in treating SAD. A randomized controlled study. BMC Psychiatry. 2011;11:17. 21276222 10.1186/1471-244X-11-17 PMC3042929

[24] Wu X , Wiater MF , Ritter S . NPAS2 deletion impairs responses to restricted feeding but not to metabolic challenges. Physiol Behav. 2010;99(4):466‐471. 20026146 10.1016/j.physbeh.2009.12.010 PMC2826533

[25] Thompson CL , Sancar A . Photolyase/cryptochrome blue‐light photoreceptors use photon energy to repair DNA and reset the circadian clock. Oncogene. 2002;21(58):9043‐9056. 12483519 10.1038/sj.onc.1205958

[26] Pohl H . Spectral composition of light as a Zeitgeber for birds living in the high arctic summer. Physiol Behav. 1999;67(3):327‐337. 10497949 10.1016/s0031-9384(99)00070-0

[27] Nishide S , Suzuki Y , Ono D , Honma S , Honma KI . The food‐entrainable oscillator is a complex of non‐SCN activity bout oscillators uncoupled from the SCN circadian pacemaker. J Biol Rhythm. 2021;36(6):575‐588. 10.1177/07487304211047937 34634956

[28] Calligaro H , Coutanson C , Najjar RP , et al. Rods contribute to the light‐induced phase shift of the retinal clock in mammals. PLoS Biol. 2019;17(3):e2006211. 30822304 10.1371/journal.pbio.2006211 PMC6415865

[29] Dudek M , Yang N , Ruckshanthi JPD , et al. The intervertebral disc contains intrinsic circadian clocks that are regulated by age and cytokines and linked to degeneration. Ann Rheum Dis. 2017;76(3):576‐584. 27489225 10.1136/annrheumdis-2016-209428 PMC5446006

[30] Yoshikawa T , Matsuno A , Yamanaka Y , Nishide SY , Honma S , Honma KI . Daily exposure to cold phase‐shifts the circadian clock of neonatal rats in vivo. Eur J Neurosci. 2013;37(3):491‐497. 23167790 10.1111/ejn.12052

[31] Smith MR , Eastman CI . Phase delaying the human circadian clock with blue‐enriched polychromatic light. Chronobiol Int. 2009;26(4):709‐725. 19444751 10.1080/07420520902927742 PMC2828683

[32] He X , Yang S , Deng J , Wu Q , Zang WJ . Amelioration of circadian disruption and calcium‐handling protein defects by choline alleviates cardiac remodeling in abdominal aorta coarctation rats. Lab Investig. 2021;101(7):878‐896. 33649466 10.1038/s41374-021-00578-6

[33] Perez‐Leighton CE , Schmidt TM , Abramowitz J , Birnbaumer L , Kofuji P . Intrinsic phototransduction persists in melanopsin‐expressing ganglion cells lacking diacylglycerol‐sensitive TRPC subunits. Eur J Neurosci. 2011;33(5):856‐867. 21261756 10.1111/j.1460-9568.2010.07583.x PMC3076293

[34] Sekaran S , Lall GS , Ralphs KL , et al. 2‐Aminoethoxydiphenylborane is an acute inhibitor of directly photosensitive retinal ganglion cell activity in vitro and in vivo. J Neurosci. 2007;27(15):3981‐3986. 17428972 10.1523/JNEUROSCI.4716-06.2007 PMC6672550

[35] Jeronimo R , Moraes MN , de Assis LVM , Ramos BC , Rocha T , Castrucci AML . Thermal stress in Danio rerio: a link between temperature, light, thermo‐TRP channels, and clock genes. J Therm Biol. 2017;68(Pt A):128‐138. 28689714 10.1016/j.jtherbio.2017.02.009

[36] Li SK , Banerjee J , Jang C , Sehgal A , Stone RA , Civan MM . Temperature oscillations drive cycles in the activity of MMP‐2,9 secreted by a human trabecular meshwork cell line. Invest Ophthalmol Vis Sci. 2015;56(2):1396‐1405. 25655795 10.1167/iovs.14-15834 PMC4340433

[37] Jeong KY , Seong J . Neonatal capsaicin treatment in rats affects TRPV1‐related noxious heat sensation and circadian body temperature rhythm. J Neurol Sci. 2014;341(1‐2):58‐63. 24746025 10.1016/j.jns.2014.03.054

[38] He Y , Wu X , Khan RS , et al. IL‐15 receptor deletion results in circadian changes of locomotor and metabolic activity. J Mol Neurosci. 2010;41(2):315‐321. 20012227 10.1007/s12031-009-9319-z PMC3939616

[39] Negri L , Lattanzi R , Giannini E , Canestrelli M , Nicotra A , Melchiorri P . Bv8/Prokineticins and their receptors a new pronociceptive system. Int Rev Neurobiol. 2009;85:145‐157. 19607967 10.1016/S0074-7742(09)85011-3

[40] Mills C , McMackin M , Jaffe R , et al. Effects of the transient receptor potential vanilloid 1 antagonist A‐425619 on body temperature and thermoregulation in the rat. Neuroscience. 2008;156(1):165‐174. 18706981 10.1016/j.neuroscience.2008.06.069

[41] Iida T , Shimizu I , Nealen ML , Campbell A , Caterina M . Attenuated fever response in mice lacking TRPV1. Neurosci Lett. 2005;378(1):28‐33. 15763167 10.1016/j.neulet.2004.12.007

[42] Szelenyi Z , Hummel Z , Szolcsányi J , Davis JB . Daily body temperature rhythm and heat tolerance in TRPV1 knockout and capsaicin pretreated mice. Eur J Neurosci. 2004;19(5):1421‐1424. 15016100 10.1111/j.1460-9568.2004.03221.x

[43] Reimundez A , Fernández‐Peña C , Ordás P , et al. The cold‐sensing ion channel TRPM8 regulates central and peripheral clockwork and the circadian oscillations of body temperature. Acta Physiol (Oxf). 2023;237(3):e13896. 36251565 10.1111/apha.13896

[44] Benedetti M , Maierová L , Cajochen C , Scartezzini JL , Münch M . Optimized office lighting advances melatonin phase and peripheral heat loss prior bedtime. Sci Rep. 2022;12(1):4267. 35277539 10.1038/s41598-022-07522-8 PMC8917232

[45] Yadlapalli S , Jiang C , Bahle A , Reddy P , Meyhofer E , Shafer OT . Circadian clock neurons constantly monitor environmental temperature to set sleep timing. Nature. 2018;555(7694):98‐102. 29466329 10.1038/nature25740

[46] Bedont JL , LeGates TA , Buhr E , et al. An LHX1‐regulated transcriptional network controls sleep/wake coupling and thermal resistance of the central circadian clockworks. Curr Biol. 2017;27(1):128‐136. 28017605 10.1016/j.cub.2016.11.008 PMC5269403

[47] Scantlebury M , Danek‐Gontard M , Bateman PW , et al. Seasonal patterns of body temperature daily rhythms in group‐living cape ground squirrels Xerus inauris. PLoS ONE. 2012;7(4):e36053. 22558324 10.1371/journal.pone.0036053 PMC3338621

[48] Vollenweider S , Wirz‐Justice A , Flammer J , Orgül S , Kräuchi K . Chronobiological characterization of women with primary vasospastic syndrome: body heat loss capacity in relation to sleep initiation and phase of entrainment. Am J Physiol Regul Integr Comp Physiol. 2008;294(2):R630‐R638. 18046019 10.1152/ajpregu.00609.2007

[49] Krauchi K . The thermophysiological cascade leading to sleep initiation in relation to phase of entrainment. Sleep Med Rev. 2007;11(6):439‐451. 17764994 10.1016/j.smrv.2007.07.001

[50] Kondo M , Tokura H , Wakamura T , et al. Combined influences of gradual changes in room temperature and light around dusk and dawn on circadian rhythms of core temperature, urinary 6‐hydroxymelatonin sulfate and waking sensation just after rising. Coll Antropol. 2007;31(2):587‐593. 17847944

[51] Wexler DB , Moore‐Ede MC . Resynchronization of circadian sleep‐wake and temperature cycles in the squirrel monkey following phase shifts of the environmental light‐dark cycle. Aviat Space Environ Med. 1986;57(12 Pt 1):1144‐1149. 3800813

[52] Kuwabara N , Seki K , Aoki K . Circadian, sleep and brain temperature rhythms in cats under sustained daily light‐dark cycles and constant darkness. Physiol Behav. 1986;38(2):283‐289. 3797494 10.1016/0031-9384(86)90164-2

[53] Asher G , Schibler U . Crosstalk between components of circadian and metabolic cycles in mammals. Cell Metab. 2011;13(2):125‐137. 21284980 10.1016/j.cmet.2011.01.006

[54] Demarque M , Schibler U . Shedding new light on circadian clocks. elife. 2013;2:e00659. 23580350 10.7554/eLife.00659 PMC3622228

[55] Morf J , Schibler U . Body temperature cycles: gatekeepers of circadian clocks. Cell Cycle. 2013;12(4):539‐540. 23343768 10.4161/cc.23670 PMC3594249

[56] Schibler U , Gotic I , Saini C , et al. Clock‐talk: interactions between central and peripheral circadian oscillators in mammals. Cold Spring Harb Symp Quant Biol. 2015;80:223‐232. 26683231 10.1101/sqb.2015.80.027490

[57] Poletini MO , Moraes MN , Ramos BC , Jerônimo R , Castrucci AML . TRP channels: a missing bond in the entrainment mechanism of peripheral clocks throughout evolution. Temperature (Austin). 2015;2(4):522‐534. 27227072 10.1080/23328940.2015.1115803 PMC4843991

[58] Warren EJ , Allen CN , Brown RL , Robinson DW . Intrinsic light responses of retinal ganglion cells projecting to the circadian system. Eur J Neurosci. 2003;17(9):1727‐1735. 12752771 10.1046/j.1460-9568.2003.02594.x PMC2435209

[59] Yang Z , Emerson M , Su HS , Sehgal A . Response of the timeless protein to light correlates with behavioral entrainment and suggests a nonvisual pathway for circadian photoreception. Neuron. 1998;21(1):215‐223. 9697865 10.1016/s0896-6273(00)80528-0

[60] Skrzypski M , Khajavi N , Mergler S , et al. Orexin a modulates INS‐1E cell proliferation and insulin secretion via extracellular signal‐regulated kinase and transient receptor potential channels. J Physiol Pharmacol. 2016;67(5):643‐652. 28011945

[61] Roessingh S , Stanewsky R . The drosophila TRPA1 channel and neuronal circuits controlling rhythmic behaviours and sleep in response to environmental temperature. Int J Mol Sci. 2017;18(10):1‐19. 10.3390/ijms18102028 PMC5666710 28972543

[62] Gomez NC , Abriouel H , Grande MA , Pulido RP , Gálvez A . Effect of enterocin AS‐48 in combination with biocides on planktonic and sessile listeria monocytogenes. Food Microbiol. 2012;30(1):51‐58. 22265283 10.1016/j.fm.2011.12.013

[63] Wolfgang W , Simoni A , Gentile C , Stanewsky R . The Pyrexia transient receptor potential channel mediates circadian clock synchronization to low temperature cycles in Drosophila melanogaster. Proc Biol Sci. 2013;280(1768):20130959. 23926145 10.1098/rspb.2013.0959 PMC3757961

[64] Yonemitsu T , Kuroki C , Takahashi N , et al. TRPA1 detects environmental chemicals and induces avoidance behavior and arousal from sleep. Sci Rep. 2013;3:3100. 24172941 10.1038/srep03100 PMC3813927

[65] Lazopulo S , Lazopulo A , Baker JD , Syed S . Daytime colour preference in drosophila depends on the circadian clock and TRP channels. Nature. 2019;574(7776):108‐111. 31534223 10.1038/s41586-019-1571-y

[66] Kimura Y , Honda M , Sasaki R , et al. The circadian rhythm of bladder clock genes in the spontaneously hypersensitive rat. PLoS ONE. 2019;14(7):e0220381. 31344120 10.1371/journal.pone.0220381 PMC6658119

[67] Ihara T , Nakamura Y , Mitsui T , et al. Intermittent restraint stress induces circadian misalignment in the mouse bladder, leading to nocturia. Sci Rep. 2019;9(1):10069. 31296902 10.1038/s41598-019-46517-w PMC6624370

[68] Ihara T , Mitsui T , Nakamura Y , et al. Clock genes regulate the circadian expression of Piezo1, TRPV4, Connexin26, and VNUT in an ex vivo mouse bladder mucosa. PLoS ONE. 2017;12(1):e0168234. 28060940 10.1371/journal.pone.0168234 PMC5218463

[69] Roessingh S , Rosing M , Marunova M , et al. Temperature synchronization of the Drosophila circadian clock protein PERIOD is controlled by the TRPA channel PYREXIA. Commun Biol. 2019;2:246. 31286063 10.1038/s42003-019-0497-0 PMC6602953

[70] Bellemer A . Thermotaxis, circadian rhythms, and TRP channels in Drosophila. Temperature (Austin). 2015;2(2):227‐243. 27227026 10.1080/23328940.2015.1004972 PMC4843867

[71] Das A , Holmes TC , Sheeba V . dTRPA1 in non‐circadian neurons modulates temperature‐dependent rhythmic activity in Drosophila melanogaster. J Biol Rhythm. 2016;31(3):272‐288. 10.1177/0748730415627037 26868037

[72] Yang SC , Chen CL , Yi CH , Liu TT , Shieh KR . Changes in gene expression patterns of circadian‐clock, transient receptor potential Vanilloid‐1 and nerve growth factor in inflamed human esophagus. Sci Rep. 2015;5:13602. 26337663 10.1038/srep13602 PMC4559770

[73] Green EW , O'Callaghan EK , Hansen CN , et al. Drosophila circadian rhythms in seminatural environments: summer afternoon component is not an artifact and requires TrpA1 channels. Proc Natl Acad Sci USA. 2015;112(28):8702‐8707. 26124142 10.1073/pnas.1506093112 PMC4507215

[74] Lee Y . Contribution of drosophila TRPA1‐expressing neurons to circadian locomotor activity patterns. PLoS ONE. 2013;8(12):e85189. 24367706 10.1371/journal.pone.0085189 PMC3867552

[75] Lee Y , Montell C . Drosophila TRPA1 functions in temperature control of circadian rhythm in pacemaker neurons. J Neurosci. 2013;33(16):6716‐6725. 23595730 10.1523/JNEUROSCI.4237-12.2013 PMC3664735

[76] Atkinson SE , Maywood ES , Chesham JE , et al. Cyclic AMP signaling control of action potential firing rate and molecular circadian pacemaking in the suprachiasmatic nucleus. J Biol Rhythm. 2011;26(3):210‐220. 10.1177/0748730411402810 21628548

[77] Chen SK , Ko GY , Dryer SE . Somatostatin peptides produce multiple effects on gating properties of native cone photoreceptor cGMP‐gated channels that depend on circadian phase and previous illumination. J Neurosci. 2007;27(45):12168‐12175. 17989283 10.1523/JNEUROSCI.3541-07.2007 PMC6673241

[78] Wen W , Yao Q , Chen Y , et al. Transient receptor potential canonical 5 channel is involved in the cardiac damage related to obstructive sleep apnea‐hypopnea syndrome in rats. Ann Palliat Med. 2020;9(3):895‐902. 32434348 10.21037/apm.2020.04.08

[79] Khalyfa A , Gozal D , Chan WC , Andrade J , Prasad B . Circulating plasma exosomes in obstructive sleep apnoea and reverse dipping blood pressure. Eur Respir J. 2020;55(1):1901072. 31672757 10.1183/13993003.01072-2019

[80] Tang L , Wang X , Wu J , et al. Sleep deprivation induces dry eye through inhibition of PPARalpha expression in corneal epithelium. Invest Ophthalmol Vis Sci. 2018;59(13):5494‐5508. 30658033 10.1167/iovs.18-24504

[81] Murillo‐Rodriguez E et al. Role of N‐Arachidonoyl‐serotonin (AA‐5‐HT) in sleep‐wake cycle architecture, sleep homeostasis, and neurotransmitters regulation. Front Mol Neurosci. 2017;10:152. 28611585 10.3389/fnmol.2017.00152 PMC5447686

[82] MacWilliam D , Arensburger P , Higa J , Cui X , Adams ME . Behavioral and genomic characterization of molt‐sleep in the tobacco hornworm, Manduca sexta. Insect Biochem Mol Biol. 2015;62:154‐167. 25661727 10.1016/j.ibmb.2015.01.012

[83] Durgan DJ , Crossland RF , Lloyd EE , Phillips SC , Bryan RM . Increased cerebrovascular sensitivity to endothelin‐1 in a rat model of obstructive sleep apnea: a role for endothelin receptor B. J Cereb Blood Flow Metab. 2015;35(3):402‐411. 25425077 10.1038/jcbfm.2014.214 PMC4348382

[84] Zhao J , Xu W , Yun F , et al. Chronic obstructive sleep apnea causes atrial remodeling in canines: mechanisms and implications. Basic Res Cardiol. 2014;109(5):427. 25015734 10.1007/s00395-014-0427-8

[85] Wei H , Gong N , Huang JL , et al. Spinal D‐amino acid oxidase contributes to mechanical pain hypersensitivity induced by sleep deprivation in the rat. Pharmacol Biochem Behav. 2013;111:30‐36. 23958579 10.1016/j.pbb.2013.08.003

[86] Donlea JM , Thimgan MS , Suzuki Y , Gottschalk L , Shaw PJ . Inducing sleep by remote control facilitates memory consolidation in drosophila. Science. 2011;332(6037):1571‐1576. 21700877 10.1126/science.1202249 PMC4064462

[87] George R , Stanewsky R . Peripheral sensory organs contribute to temperature synchronization of the circadian clock in Drosophila melanogaster. Front Physiol. 2021;12:622545. 33603678 10.3389/fphys.2021.622545 PMC7884628

[88] Li K , Shi Y , Gonye EC , Bayliss DA . TRPM4 contributes to subthreshold membrane potential oscillations in multiple mouse pacemaker neurons. eNeuro. 2021;8(6):ENEURO.0212‐21.2021. 10.1523/ENEURO.0212-21.2021 PMC8607911 34732535

